# Batokines: Mediators of Inter-Tissue Communication (a Mini-Review)

**DOI:** 10.1007/s13679-021-00465-7

**Published:** 2022-01-07

**Authors:** Felix T. Yang, Kristin I. Stanford

**Affiliations:** 1 Department of Physiology and Cell Biology, The Ohio State University Wexner Medical Center, 460 W. 12th Ave, Columbus, OH 43210 USA; 2 Diabetes and Metabolism Research Center, Dorothy M. Davis Heart and Lung Research Institute, The Ohio State University Wexner Medical Center, Columbus, OH USA; 3 Department of Internal Medicine, The Ohio State University Wexner Medical Center, Columbus, OH USA

**Keywords:** Brown adipose tissue, Endocrine, Metabolism, Batokines, Obesity, Inter-tissue communication, Paracrine

## Abstract

***Purpose of Review*:**

This review highlights aspects of brown adipose tissue (BAT) communication with other organ systems and how BAT-to-tissue cross-talk could help elucidate future obesity treatments.

***Recent Findings*:**

Until recently, research on BAT has focused mainly on its thermogenic activity. New research has identified an endocrine/paracrine function of BAT and determined that many BAT-derived molecules, termed “batokines,” affect the physiology of a variety of organ systems and cell types. Batokines encompass a variety of signaling molecules including peptides, metabolites, lipids, or microRNAs. Recent studies have noted significant effects of batokines on physiology as it relates whole-body metabolism and cardiac function. This review will discuss batokines and other BAT processes that affect the liver, cardiovascular system, skeletal muscle, immune cells, and brown and white adipose tissue.

***Summary*:**

Brown adipose tissue has a crucial secretory function that plays a key role in systemic physiology.

## Introduction

The prevalence of obesity has risen remarkably over the last few decades; with over 50% of the world’s population being classified as overweight or having obesity, the need to identify therapeutic tools to treat obesity is of paramount importance [1]. Obesity is a disease of disproportionate body fat due to excessive weight gain. One of the most important tissues in mediating the metabolic derangements of obesity is adipose tissue [2].

There are three types of adipose tissues in rodents and humans: white adipose tissue (WAT), brown adipose tissue (BAT), and beige (also known as brite) adipose tissue. WAT is composed of unilocular adipocytes which act as depots to store energy in the form of triglycerides and release them during fasting, physical activity, or other energy expenditure states [3]. Conversely, BAT is composed of multi-locular and mitochondrial-rich adipocytes which are involved in energy expenditure and play a role in non-shivering thermogenesis [4]. Beige adipose “tissue” in rodents is composed of “brown-like” or beige adipocytes interspersed within white adipose tissue [5]. These beige adipocytes are adipocytes that underwent white-to-brown transitioning within WAT depots and share phenotypic similarities to brown adipocytes (i.e., thermogenic capacity, multi-locular lipid droplets). In this sense, beige adipocytes developmentally differ from brown adipocytes due to their inducible nature from white adipocytes, whereas brown adipocytes actually arise from a shared precursor with skeletal myocytes [6]. While these cellular and molecular distinctions between brown and beige adipocytes have been described in rodents [5, 6], the actual classification of human thermogenic adipose tissue as “brown” vs “beige” remains a topic of debate (see the review by Cannon et al. [7] for further discussion). However, for the purposes of this review, human thermogenic adipose tissue will be referred to as *brown* adipose tissue or BAT.

An emerging body of research suggests that the physiological functions of BAT extend beyond thermogenesis and that BAT is actually an endocrine organ. The molecular mediators of these endocrine effects of BAT are termed “batokines.” Batokines are BAT-derived molecules that affect the physiological function of organ systems and can signal to a variety of cell types. This review will examine the role of batokines and other BAT mechanisms that mediate BAT’s endocrine functions on systemic metabolism, cardiac physiology, and more.

## Functions of BAT in Normal and Pathophysiological Conditions

BAT has a variety of functions but has canonically been described as the primary source of non-shivering thermogenesis in response to cold [8, 9]. One of the most-studied effectors in BAT thermogenesis is UCP1 [10] which is a protein that functions to uncouple oxidative phosphorylation in the mitochondria via a leaky proton channel [11]. Thermogenic activity in BAT results in the consumption of fuels such as glucose and fatty acids [12, 13]—suggesting that BAT also plays an important functional role in whole-body metabolism. In fact, thermogenic activation of BAT via cold exposure has been demonstrated to increase glucose uptake in BAT and improve whole body insulin sensitivity in patients with obesity or type 2 diabetes [14, 15]. BAT activation upon cold exposure is due to increased sympathetic outflow of norepinephrine [16] which primarily acts on β3-adrenergic receptors. While sympathetic β3-adrenergic signaling is not the sole pathway involved in BAT thermogenic activation—there are other signalling molecules (i.e., thyroid hormones and leptin) that coordinate with the sympathetic nervous system to induce BAT thermogenesis—it is certainly the most well studied [17]. In addition to increased BAT thermogenesis, β3-activation of BAT increases lipolysis [18] and insulin sensitivity [19], indicating that β-adrenergic stimulation of BAT has metabolic consequences.

These findings have led some to postulate that increasing BAT mass through transplantation (rather than sympathetic stimulation of BAT) improved glucose metabolism and insulin sensitivity [12, 20]. In one of these BAT transplantation studies [12], our lab observed that BAT transplantation increased circulating IL-6, FGF21, and norepinephrine concentrations. The results of these BAT transplantation studies indicate that BAT has paracrine or endocrine functions that leads to the release of secreted factors (known as batokines) that could be mediating these metabolic improvements. And although it is unclear whether innervation or vascularization of the donor BAT preceded the metabolic changes in the recipient, the absence of thermogenic stimuli and the changes in certain secreted factors in these transplant experiments suggest that BAT plays a paracrine/endocrine function in systemic physiology. This idea is supported by other studies that have shown that pharmacological or cold stimulation leads to a release of batokines concurrent with activation of BAT thermogenesis [21, 22]. However, the degree of coordination between BAT’s thermogenic activity and BAT’s endocrine activity and their respective roles in whole-body physiology remains an open question.

In the context of obesity, BAT mass and activity (as defined by ^18^F-FDG PET/CT) is severely reduced, which could be a contributing factor to the development of obesity and obesity-associated disorders such as type 2 diabetes [23–25]. While it is not clear whether this reduction in BAT mass and activity is a cause or consequence of obesity, the beneficial role of BAT and thermogenic adipose tissue in obesity and metabolic disease has been corroborated by several studies. One of these studies showed that the use of mirabegron, a β3-agonist, led to improvements in glucose tolerance and insulin sensitivity in humans having obesity [26]. Because the β3-adrenergic receptor is more abundantly expressed in adipose tissue than other tissues [27], the beneficial metabolic effects of mirabegron in this study could be due to BAT activation or white adipose tissue “beiging.”

While the metabolic effects of BAT are well-characterized, a recent study showed that BAT has a protective role against obesity-related cardiometabolic disease [25]. In this retrospective study, individuals with active BAT (confirmed via ^18^F-FDG PET/CT) had a lower propensity of obesity-related cardiometabolic diseases such as congestive heart failure, hypertension, and dyslipidemia [25]. In this same study, the inverse association between increased BAT activity and reduced cardiometabolic disease was more pronounced in individuals having obesity, indicating that active BAT can protect against the cardiovascular and metabolic comorbidities of obesity in humans. It is important to note that the study by Becher et al. did not identify any of the potential physiologic mechanisms by which BAT improves these cardiometabolic outcomes in humans. One potential mechanism could be due to BAT’s function in energy expenditure and its putative role in improving glucose and lipid metabolism [14, 28]. However, several studies have demonstrated that other organs/tissues such as the heart and skeletal muscle play a greater role in overall energy expenditure and the clearance of glucose and lipid-related substrates [29, 30], thus suggesting that BAT’s beneficial effects in humans may be mediated by its endocrine/paracrine effects on other tissues.

**Fig. 1:**
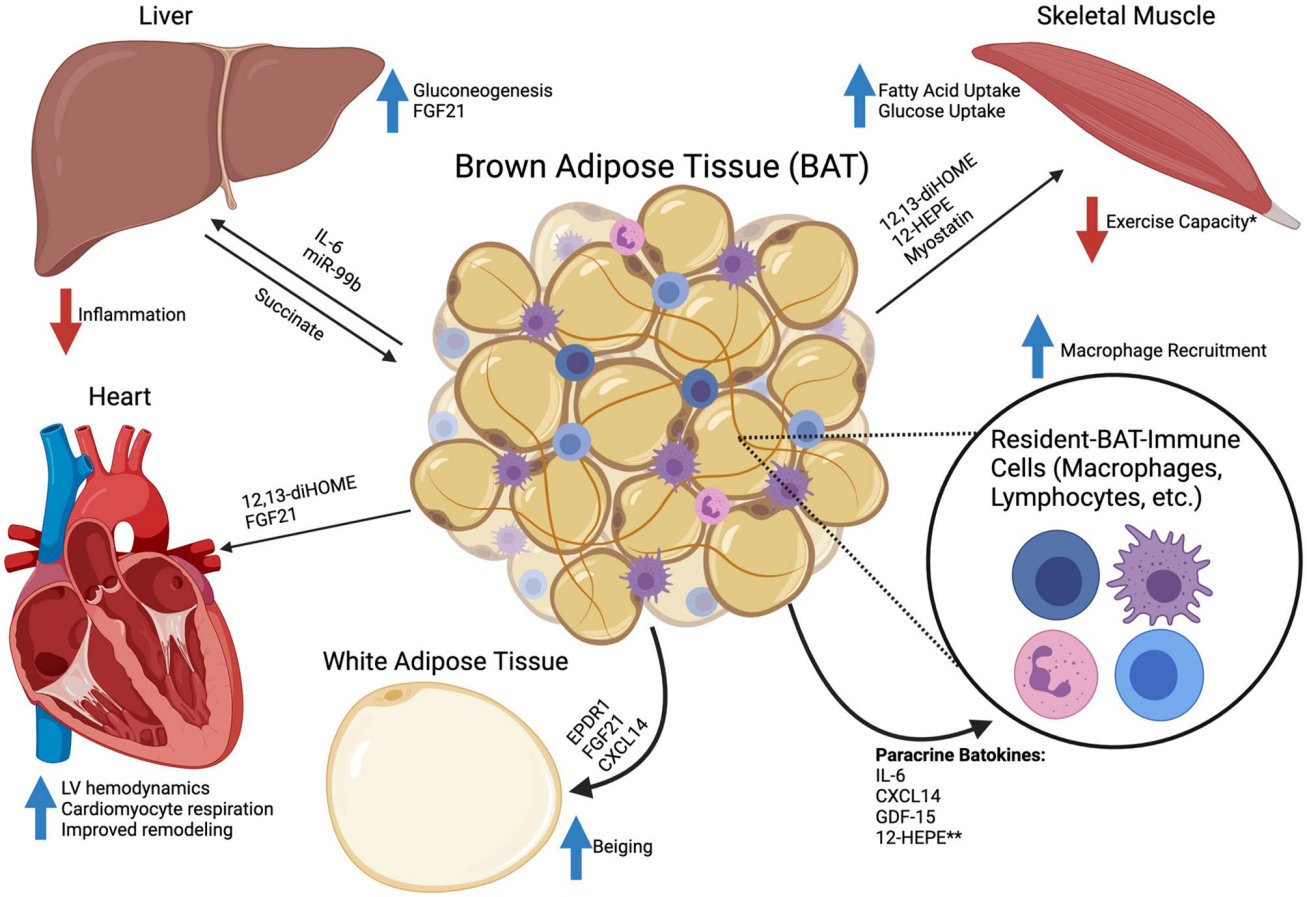
Brown adipose tissue inter-tissue and inter-cellular communication. Mechanisms of brown adipose tissue (BAT) bi-directional tissue and cellular communication. Batokines (secreted factors from BAT) target organs such as the white adipose tissue, liver, skeletal muscle, and heart. Additionally, many paracrine batokines target immune cells with the most well-studied effects on macrophages. *The reduction in exercise capacity is due to secretion of myostatin from BAT at thermoneutral conditions. **No study has specifically shown an effect of 12-HEPE on resident-BAT immune cells; the effects of BAT-derived lipokines on local immune cells is still unknown. Created with BioRender.com

As such, the previously mentioned studies, along with several other mouse and human studies covered in this review, suggest that BAT’s function in physiology is not merely in energy expenditure and thermogenesis but that it also has a secretory function that affects many other organ systems. The remainder of this mini-review will focus on the secretory role of batokines and other mechanisms of inter-cellular and inter-tissue communication between BAT and a variety of organ systems and cell types (Fig. 1).

## BAT-and-Liver Crosstalk: Regulation of Whole-Body Metabolism

Among the first batokines discovered was FGF21, where it was shown to be secreted from BAT upon thermogenic activation [31]. FGF21 secretion is not exclusive to BAT; it is secreted primarily from the liver and is a key regulator of metabolic function due to its pleiotropic effects on multiple different tissues including the heart, skeletal muscle, brain, and the liver itself [32]. In terms of the specific effects of FGF21 on the liver, many studies have indicated that FGF21 could protect against the development of steatosis and nonalcoholic fatty liver disease [33]. As for the effects of FGF21 on BAT, a study by Emanuelli et al. showed that FGF21 treatment in LIRKO mice (a liver-specific insulin receptor knockout model) increased glucose uptake in BAT and browning of WAT [34]. Interestingly, in that same study, removal of interscapular BAT (the largest BAT depot in mice) did not impede the metabolic consequences of FGF21 administration—leading the authors to conclude that remaining BAT depots or browning of WAT were sufficient to mediate the effects of FGF21. The above studies indicate that FGF21 has effects on BAT and liver physiology, but it is unknown whether BAT-derived FGF21 or liver-derived FGF21 are mediators of bi-directional communication between the two organs.

While thermogenic activation of BAT induces BAT FGF21 secretion [31], somewhat paradoxically, UCP1-knockout mice also have increased plasma FGF21 at sub-thermoneutral temperatures [35]. Given the well-described role of UCP1 in BAT thermogenesis, one might expect for UCP1-KO mice to have impaired BAT thermogenic function and, consequently, a *decrease* in FGF21 levels. However, the finding that UCP1-dependent thermogenesis is not specifically tied to BAT secretion of FGF21 suggests that BAT thermogenic function might not be specifically tied to its endocrine function. Other studies have showed that non-thermogenic modalities of increasing BAT activity and mass, through methods like BAT transplantation [12], also lead to an increase serum FGF21. These findings along with other recent studies could suggest that the secretion of BAT-derived FGF21 and its subsequent metabolic effects may not be specifically linked to BAT thermogenic activity [36].

Another batokine that has been demonstrated to signal to the liver is IL-6 [12]. Similar to FGF21, β-adrenergic stimuli [37] as well as transplantation [12] increases BAT-derived IL-6. In a study by Stanford et al., BAT transplantation from IL-6 knockout mice did not induce any of the typical metabolic improvements seen with BAT transplantation. A study by Qing et al. showed that acute psychological stress led to the release of IL-6 from BAT and thereby enhanced hepatic gluconeogenesis [38] as a part of a “fight or flight” response. Interestingly, while the previous studies have shown that IL-6 plays a key role in the metabolic homeostatic effects of BAT, Qing et al. demonstrated that the increase in IL-6 was independent of the BAT thermogenic program, reiterating that the release of batokines that regulate systemic metabolism are independent of the classical descriptions of BAT activation (i.e., thermogenesis).

While the aforementioned research has focused on the peptide class of batokines, microRNAs (miRNA) have been noted as a mediator of BAT-to-liver endocrine signaling. A number of adipose-derived miRNAs have been detected in extracellular vesicles and contribute to the regulation of systemic metabolism [39]. In a mouse model of adipose-specific deficient microRNA processing (ADicerKO), BAT transplantation restored the levels of circulating miRNAs and was the only type of adipose tissue transplant (compared to WAT) to significantly improve glucose tolerance in the ADicerKO mice [40]. Follow-up analysis within the same study identified that miR-99b was the key microRNA that was increased upon BAT transplantation and specifically regulated hepatic production of FGF21. Given that it was recently reported that hepatic FGF21 signaling upregulates BAT thermogenesis [41], it is possible that thermogenic release of BAT-derived miRNAs on hepatic FGF21 release acts as a reciprocal mechanism in BAT-liver crosstalk to maintain core body temperature. To date of this review article, there are few other studies that describe the role of exosomal microRNAs released from BAT [42]—which demonstrates the need for further investigation into this topic.

Finally, another consideration of how BAT can affect liver function is through its ability to modulate extracellular metabolite concentration. Mills et al. showed that UCP1^+^ adipocytes were responsible for the clearance and catabolism of liver extracellular succinate [43]. In this study, they observed that UCP1KO mice on an obesogenic diet exhibited increased levels of extracellular succinate in the liver and had elevated levels of liver inflammation. After making this observation, Mills et al. conducted a series of isotope-tracing experiments to demonstrate that acute activation of BAT via β3-adrenergic-agonist treatment or cold exposure directly increased succinate uptake by BAT and its resulting catabolism. They concluded that UCP1 activity in brown/beige fat regulated liver extracellular succinate and was a key regulator in liver inflammatory pathogenesis. This recognition that BAT can regulate systemic physiology due to metabolite handling opens up new avenues of research for BAT’s role as an endocrine tissue.

## BAT-to-Heart Batokines: the Role of Batokines in Cardiovascular Function

The relationship between brown adipose tissue and cardiovascular disease can be traced back to 20 years ago when it was shown that murine brown adipose tissue ablation resulted in a number of pathologic cardiovascular outcomes such as systemic hypertension and cardiac fibrosis [44]. While there have been several studies that have reaffirmed the relationship between BAT and cardiac function in mice [45, 46], current literature on batokines affecting the cardiovascular system remains relatively unexplored.

One batokine that has recently been described to enhance cardiac function is 12,13 diHOME (12,13-dihydroxy-9Z-octadecenoic acid) an oxidized linoleic acid metabolite (oxylipin) [47]. By utilizing a BAT transplantation model, our lab showed that 12,13-diHOME, a lipokine released from BAT, increased cardiac function (improved left ventricular hemodynamics) and cardiomyocyte respiration via enhanced calcium cycling. In addition to BAT transplantation, the increases in 12,13-diHOME have been seen with both cold exposure and exercise training [48, 49]. Indeed, this was the first study of its kind to demonstrate a direct effect by a BAT-derived lipokine on the heart and points to the importance for further research into lipid-derived batokines.

Although the role of BAT-derived FGF21 in metabolic homeostasis has been thoroughly characterized [31], the role of BAT-derived FGF21 on the heart has been less well described. A study by Ruan et al. [50] demonstrated that FGF21 from BAT regulated hypertensive cardiac remodeling. In this study, they showed that catecholamine-driven activation of BAT via a A_2A_R-dependent mechanism led to the release of FGF21 and protected against pathologic hypertensive cardiac remodeling.

The aforementioned studies indicate that the role of BAT in physiologic cardiac function is still a topic of open investigation. In fact, it was only recently confirmed that there was an inverse association between the presence of BAT and cardiometabolic disease in humans [25]. In this study, Becher et al. reviewed ^18^F-FDG PET/CT reports of over 50,000 patients and found that presence of BAT indicated a lower risk for cardiometabolic diseases. Among such cardiometabolic diseases included type 2 diabetes, dyslipidemia, coronary artery disease, cerebrovascular disease, congestive heart failure, and hypertension. Additionally, a number of mouse studies and recent clinical studies have indicated that BAT activation via cold-exposure or pharmacological agents led to improvements in cholesterol levels, HDL levels, and a reduction in atherosclerotic progression [19, 51, 52]. The mechanisms that link BAT and cardiometabolic health remain largely unknown and it is likely that batokines that affect cardiac and vascular tissues are somehow involved. In summary, these findings indicate the need for further investigation into batokines that mediate cardiovascular function.

## Exercise-Associated Batokines: Effectors of Skeletal Muscle Physiology

The most well-studied contribution of physical exercise to overall physiological health is through exercise-induced adaptations to skeletal muscle. However, exercise in rodents and humans has also been shown to cause numerous adaptations to white adipose tissue [53]. In mice, WAT adaptations due to exercise result in increased WAT thermogenic capacity—referred to as “beiging”—and improvements in glucose homeostasis [54], whereas several human studies have determined that exercise does not induce beiging of WAT [55, 56]. While exercise-induced beiging of WAT causes it to take on many similar attributes to BAT, the relationship between physiological BAT function and exercise remains a topic of open investigation. In mice, exercise has been reported to have conflicting results in the thermogenic activation of BAT [57, 58], whereas in humans, exercise has been shown to decrease BAT glucose uptake [59, 60]. Since exercise itself is a thermogenic activity, one might expect to have a *decrease* in BAT thermogenic activity and consequently a *decrease* in the beneficial effects related to its endocrine function. However, work in our lab has identified that exercise can also alter the endocrine function of BAT for changed metabolic outcomes on other tissues [48]. Of these findings, we observed that exercise increased the secretion of 12,13-diHOME to increase fatty-acid uptake in skeletal muscle and that removal of BAT (in mice) nullified the exercise-induced increases in 12,13-diHOME. The finding that exercise alters the secretion of 12,13-diHOME from BAT illustrates that beneficial BAT secreted activity can occur in the context of non-thermogenic states and challenges the convention of assessing BAT thermogenesis as the only means of examining BAT activity. Further discussion for the rest of this section will speculate on how certain BAT stimuli such as exercise may affect BAT endocrine function in skeletal muscle.

As mentioned earlier, previous research from our lab and others have identified that 12,13-diHOME is a lipokine from BAT that is released in response to cold and exercise in mice and humans [47–49]. While this review has already covered the cardiac effects of 12,13-diHOME, 12,13-diHOME also effects skeletal muscle [48]. Acute treatment of 12,13-diHOME in mice increased skeletal muscle fatty acid uptake and oxidation, but not glucose uptake. In contrast, another batokine, 12-HEPE, improved glucose metabolism via increased glucose uptake into adipocytes and skeletal muscle [21]. It is important to note that in this study, 12-HEPE secretion by BAT was only shown to be activated due to cold or β3-adrenergic stimulation whereas the effect of exercise training on the secretion of 12-HEPE from BAT is unchanged [21].

Another batokine known to be associated with exercise is myostatin (also known as GDF-8) [61]. Kong et al. observed that myostatin was released by BAT and reduced the exercise capacity of mice. More specifically, they observed that BAT-specific loss of the transcription factor IRF4 led to elevated myostatin and resulted in reduced exercise capacity, abnormalities of the white vastus muscle, and aberrant mitochondrial function within the myocytes. Interestingly, the release of myostatin from BAT was *increased* at thermoneutrality, which is typically described as a “BAT-inactive” state in terms of thermogenesis. While this finding affirms the notion that increasing BAT thermogenesis may lead to more “positive” physiological outcomes for the organism (i.e., improved exercise capacity), it also disputes the notion that BAT is “inactive” at thermoneutrality and demonstrates that BAT is capable of affecting other organs via secreted factors during thermogenically inactive states. Again, such distinctions should be made by researchers when describing BAT activity as it relates to its thermogenic vs endocrine function. Furthermore, the secretion of myostatin by BAT illustrates how batokines can act on skeletal muscle to enable complementary physiologic processes (i.e., non-shivering thermogenesis and shivering thermogenesis) to achieve a synergistic functional relationship.

## Paracrine Batokines: Regulators of Brown Adipose Tissue Immunity

A large number of batokines have been described in terms of their autocrine and paracrine function. Many of BAT’s autocrine/paracrine-secreted factors function to facilitate the thermogenic function of BAT through actions on the adipocyte itself, or on local endothelial, neuronal, or immune cells [62, 63]. In the context of obesity, BAT releases an altered millieu of autocrine/paracrine batokines due to an increased presence of pro-inflammatory immune cells within the BAT depot [64]. These BAT-resident immune cells have been shown to release molecules (Oncostatin M, IL-1β) that can lead to an impairment in BAT’s thermogenic function and/or UCP1 expression [65, 66]. While these topics are extensively covered here [62–64], this mini-review will provide a brief perspective on how certain batokines and other secreted factors from BAT regulate immune cell function.

A number of batokines have been canonically identified to have a role in the immune system’s function. As mentioned earlier, BAT-derived IL-6 regulates hepatic gluconeogenesis [38]. However, IL-6 has classically been described as a key signaling molecule in the formation of a pro-inflammatory response [67]. While there has been little research done to define the role of BAT-derived IL-6 in the function of the immune system or resident-adipose-tissue immune cells, one study has shown that the tissue-specific source of IL-6 (whether from myeloid, myocytes, or adipocytes) led to differential outcomes when it came to adipose tissue macrophage accumulation [68]. Of the outcomes, white adipose tissue-derived IL-6 led to increased macrophage accumulation whereas myeloid and muscle-derived IL-6 suppressed macrophage accumulation within white adipose tissue. The role of BAT-derived IL-6 on macrophages remains unclear and further investigation on the effects of BAT-derived IL6 on different immune cell types is warranted.

CXCL14 is a batokine that has a role in macrophage accumulation in BAT [69]. Cereijo et al. identified CXCL14 as a batokine that is released upon thermogenic activation and is important to M2 macrophage recruitment within different adipose tissue depots. It is interesting to note from this study that M2 macrophage recruitment due to CXCL14 occurred concertedly with thermogenic activation of BAT and browning of WAT. CXCL14 is not the only batokine reported to have effects on macrophages; GDF-15 is another batokine that targets macrophages and downregulates their proinflammatory activity in association with enhanced BAT thermogenesis [70]. Interestingly, while also being dependent on noradrenergic stimulation, the release of GDF-15 from BAT was also dependent on the induction of BAT-derived FGF21.

Finally, there are certain lipid-derived batokines such as 12-HEPE (12-hydroxyeicosapentaenoic acid) [21] and 12,13-diHOME [48, 49] that have been described as mediators of immune cell function and/or inflammation in various other tissues [71–73]. For example, 12,13,-diHOME was previously described a pro-inflammatory agent with cytotoxic properties and has also been described as an inhibitor of neutrophil respiratory burst [74, 75]. In a more recent study, Levan et al. identified that 12,13-diHOME secreted from the gut microbiome has been reported to promote allergic inflammation in neonates and alter lipid uptake in human dendritic cells [72]. Similarly, administration of 12-HEPE resulted in inhibiting the transformation of macrophages to an inflammatory foam cell phenotype [71]. This line of evidence suggests that there might be a physiological role for BAT-derived signaling lipids to modulate circulating immune cell or tissue-specific immune cell function. However, to the best of our knowledge, no study has closely examined how the release of BAT-specific secreted factors due to certain stimuli (i.e., cold, exercise) may affect immunity outside of the parent tissue. And while the aforementioned studies show that the local immune environment within BAT and WAT play a key role in these tissues thermogenic function, more research must be pursued to ascertain whether the secretion of these potential immunomodulatory factors from BAT bear any physiological consequences.

## Conclusion

BAT’s known physiological role in energy expenditure has led to a resurgence in research in targeting BAT activation or increasing BAT mass (BAT induction) as a means to increase energy expenditure to treat obesity. The recent identification of numerous batokines has redefined BAT as an endocrine, as well as thermogenic, organ. BAT has shown that it plays a role beyond non-shivering thermogenesis and energy expenditure and could be a key regulator in a variety of physiological processes such as whole-body metabolism, cardiac function, and blood pressure due to its endocrine functions. Elucidating the mechanisms by which BAT communicates inter-cellularly within the adipose depot and its bidirectional communication with other organ systems could play an important role in uncovering potential therapeutics to help treat obesity and its associated comorbidities.
